# Assessing the social and physical determinants of circumpolar population health

**DOI:** 10.3402/ijch.v72i0.21400

**Published:** 2013-08-05

**Authors:** David L. Driscoll, Bruce Dotterrer, Richard A. Brown

**Affiliations:** Institute for Circumpolar Health Studies, University of Alaska, Anchorage, USA

**Keywords:** arctic, population health, determinants, systematic review

## Abstract

**Introduction:**

Systematic reviews of the social and physical determinants of health provide metrics for evaluation of programs to mitigate health disparities. Previous meta-analyses of the population health literature have identified several proximate social and physical determinants of population health in the circumpolar north including addiction, environmental exposures, diet/nutrition and global climate change. Proximate health determinants are most amenable to early detection and modification or mitigation through disease prevention or health promotion interventions.

**Design:**

There is a need for research to replicate these findings based on the latest science. This presentation describes a study applying Dahlgren and Whitehead's (1991) socio-ecological model of health determinants to identify the proximate social and physical determinants of health in the circumpolar north.

**Methods:**

The study consisted of a systematic review of recent studies that link determinants of health with the leading causes of mortality and morbidity in Alaska. Our search strategy employed a keyword search using the Circumpolar Health Bibliographic Database (CHBD) and 4 databases within the Web of Knowledge (WoK) data gateway. Keywords included various terms for the arctic, all relevant nations and territories within the region, as well as leading health outcomes.

**Results:**

Studies meeting the following inclusion criteria were reviewed: original research within a circumpolar population, published in English during 2011, and involving a rigorous demonstration of a link between a social determinant and selected health outcomes.

**Conclusions:**

Study conclusions includes a list of determinants identified, their associated outcomes and the study designs implemented to assess that association.

Social and physical determinants of health are the social factors and physical conditions that shape whether individuals stay healthy or become ill (1). Furthermore, the social and physical determinants of health are a useful lens through which to view the health disparities that could lead to preventable health outcomes (2). In the period since the social and physical determinants of health gained recognition as a foci for health promotion programs, many important organizations have targeted specific determinants to set priorities for research and policy in both international (3) and national (4) initiatives. Some have argued, however, that these programs fail to include a circumpolar health perspective (5). Many circumpolar communities face health challenges that differ from those encountered by more southerly communities. Recent scholarship describes obstacles to accessing quality health care in geographically and socially isolated circumpolar settings (6), experiences with extraordinarily rapid economic development (7), and growing health disparities in culturally distinct and indigenous populations (8). In the field of environmental health, the Arctic has become a sentinel for issues such as the disruption of traditional ways of life due to climate change impacts (9). There is a need for further investigation into the determinants of health outcomes in the circumpolar north.

Faculty and staff at the Institute for Circumpolar Health Studies (ICHS) conducted a review to assess what determinants of circumpolar health were described in the published literature in 2011. This literature review was part of an ongoing process to inform the research portfolio at the ICHS, and contribute to the global health discourse on determinants by identifying contributions and gaps in the literature that may indicate advancements or lags in the conduct of research related to prospective determinants of circumpolar health. This review had 2 over-arching objectives:

- Identify studies that demonstrate associations, either positive or negative, between determinants of population health and circumpolar health outcomes, and
- Characterize key features of the research methodologies currently employed by researchers focusing on the circumpolar north.

This article describes the approaches employed in a review of the literature published in 2011 and compares the results of this study with a similar review of the literature published in 2009. We conclude with an assessment of what determinants of circumpolar health are addressed in recent health literature.

## Methods

Previous studies have described the limitations of electronic data archiving in searching for, and synthesizing, relevant publications during systematic reviews (10, 11). We elected to employ the Web of Knowledge (WoK) because it is a journal index that includes a large body of health literature, and also contains a means of limiting the geographic search in a way that does not include an author's geographically based affiliation while still including geographic terms relevant to the reported study's location (12).

Review criteria included original research taking place in the circumpolar north that includes a rigorous association between a social determinant and selected disease outcome. Publications were either in English or were English translations. We did not include review articles or those studies including populations outside the circumpolar north. We defined the circumpolar north as the geographic region north of 60° north latitude. Search terms for geographic keywords included English translations and English spellings of local names whenever possible. In the event of multiple translations, both versions were entered. Terms for search strings are shown in Table I.

**Table I:** Primary (geographic) and secondary (leading mortality and morbidity in Alaska) search strings used to identify reviewable research articles

Geographical areas	
	“circumpolar” or “polar” or “arctic” or “subarctic” or “sub-arctic” or “Alaska” or “Aleutian Islands” or “Canada” or “Northwest Territories” or “Yukon” or “Nunavut” or “Baffin Islands” or “Greenland” or “Iceland” or “Faroe Islands” or “Scandanavia” or “Norway” or “Svalbard” or “Finnmark” or “Tromsø” or “Tromso” or “Nordland” or “Nord-Trøndelag” or “Møre og Romsdal” or “Nord-Trondelag” or “More og Romsdal” or “Sogn og Fjordane” or “Oppland” or “Hedmark” or “Sweden” or “Norrland” or “Svealand” or “Finland” or “Lapland” or “Northern Ostrobothnia” or “Kainuu” or “Russia” or “Kaliningrad” or “Pskov” or “Leningrad” or “Novgorod” or “Smolensk” or “Bryansk” or “Kursk” or “Belograd” or “Kaluga” or “Oryol” or “Tula” or “Lipetsk” or “Voronezh” or “Moscow” or “Ryazan” or “Tambov” or “Ivanovo” or “Vladimir” or “Penza” or “Chuvashia” or “Mordovia” or “Murmansk” or “Kareliya” or “Karelia” or “Arkhangelsk” or “Nenets” or “Nenetsia” or “Vologda” or “Tver” or “Smolensk” or “Yaroslavl” or “Kirov” or “Mari El” or “Komi” or “Perm” or “Sverlovsk” or “Yamalia” or “Khanty-Mansi” or “Khantia-Mansia” or “Yugra” or “Krasnoyarsk” or “Koryak” or “Magadan” or “Norrbotten” or “Oulu” or “Sakha” or “Yakutia” or “Taymyr” or “Västerbotten” or “Yamalo-Nenets” or “Siberia” or “Kamatchka” or “Chukotka” or “Khabarovsk”
Cancer	
ICD-10: C00–C97	“Malignant neoplasms” or “cancer” or “carcinoma” or “tumor” or “tumour” or “neoplasm” or “onco” or “lymphoma” or “macroglobulinaemia” or “Franklin disease” or “gamma heavy chain disease” or “immunoprliferative disease” or “myeloma” or “leukemia” or “leukaemia” or “Kahler's” or “Hodgkins” or “Hodgkin” or “Hodgkin's” or “non-Hodgkins” or “non-Hodgkin” or “non-Hodgkin's” or “myelomatosis” or “plasmacytoma” or “sarcoma” or “Langerhans-cell histiocytosis” or “melanoma” or “mesothelioma”
Heart disease	
ICD-10: I00–I09 or I11 or I13 or I20–I51	“Heart disease” or “rheumatic” or “pericarditis” or “endocarditis” or “myocarditis” or “Rheumatic chorea” or “mitral valve disease” or “mitral stenosis” or “rheumatic mitral insufficiency” or “aortic stenosis” or “aortic insufficiency” or “rheumatic tricuspid valve disease” or “tricuspid stenosis” or “angina” or “coronary disease” or “ischemia” or “ischaemic” or “angina” or “Dressler's syndrome” or “myocardial infarction” or “hypertension” or “hypertensive” or “congestive heart failure” or “heart failure” or “hypertensive renal failure” or “high blood pressure” or “embolism” or “embolus” or “thrombosis” or “thrombus” or “atherosclerosis” or “atherosclerotic” or “heart aneurysm” or “cardiomyopathy” or “pulmonary embolism” or “pulmonale” or “kyphoscoliotic” or “arteriovenous fistula” or “haemopericardium” or “hemopericardium” or “pericardial effusion” or “pericardium” or “pulmonary valve” or “cardiomyopathy” or “endomyocardial” or “eosinophilic” or “fibroelastosis” or “atrioventricular block” or “fascicular block” or “bifascicular block” or “trifascicular block” or “bundle-branch block” or “sinoatrial block” or “sinoauricular block” or “intraventricular block” or “conduction disorder” or “paroxysmal tachycardia” or “ventricular arrhythmia” or “supraventricular tachycardia” or “atrial fibrillation” or “atrial flutter” or “atrial premature depolarization” or “sick sinus syndrome” or “cardiac arrhythmia” or “heart failure” or “left ventricular failure” or “cardiac asthma” or “oedema of the lung” or “pulmonary oedema” or “cardiac septal defect” or “rupture of papillary muscle” or “rupture of chordae tendeae” or “cardiomegaly” or “ventricular dilatation” or “carditis” or “pancarditis” or “heart attack” or “cardiovascular disease” or “CVD” or “CHD”
Unintentional injury	
ICD-10: V01-X59 or Y85Y86	“Unintentional injury” or “injury” or “wound” or “trauma” or “accident” or “falls” or “exposure to elements” or “blunt force” or “drowning” or “submersion” or “electrocution” or “burns” or “heat exposure” or “radiation exposure” or “venom” or “animal attack” or “poison” or “noxious” or “overexertion” or “privation”
Respiratory disease	
ICD-10: J40–J47	“Chronic lower respiratory disease” or “chronic obstructive pulmonary disease” or “copd” or “chronic bronchitis” or “emphysema” or “tracheitis” or “tracheobronchitis” or “emphysematous” or “MacLeod's syndrome” or “asthma” or “asthmaticus” or “bronchiolectasis”
Intentional self-injury	
ICD-10: X60-X84 or Y87.0	“Intentional self-injury” or “suicide” or “parasuicide” or “self-poisoning” or “self-harm” or “self-inflicted”
Diabetes	
ICD-10: E10–E14	“Diabetes” or “diabetic” or “diabetic coma” or “acidosis” or “ketoacidosis” or “hyperosmolar coma” or “hypoglycemic coma” or “hypoglycaemic coma” or “diabetic nephropathy” or “intracapillary glomerulonephrosis” or “Kimmelstiel-Wilson syndrome” or “neuropathy” or “glycosuria” or “hypoinsulinaemia” or “hypoinsulinemia” or “impaired glucose tolerance”
Stroke	
ICD-10: I60–I69	“Cerebrovascular disease” or “stroke” or “intracerebral” or” subarachnoid haemorrhage” or “subarchnoid hemorrhage” or “cerebral infarction” or “occlusion” or “stenosis” or “cerebral aneurism” or “cerebral thrombosis” or “cerebral embolism” or “Binsanger's disease” or “moyamoya disease” or “nonpyogenic thrombosis of intracranial venous system” or “cerebral arteritis”
Influenza and pneumonia	
ICD-10: J10–J18	“Influenza” or “pneumonia” or “pneumoniae” or “bronchopneumonia” or “influenziae” or “Legionnaires’”
Sexually transmitted infections/HIV	
ICD-10: B20–B24 or A50–A64	“HIV”or “AIDS” or “STI” or “STD” or “human immunodeficiency virus” or “sexually transmitted” or “syphilis” or “syphilitic” or “neurosyphilis” or “gonococcal” or “gonorrhea” or “chlamydia” or “chlamydial” or “chanchroid” or “ulcus molle” or “granuloma inguinale” or “donovanosis” or “herpes” or “trichomoniasis” or “herpesviral” or “molluscum contagiosum” or “papilloma” or “anogenital warts” or “genital warts” or “venereal warts”
Tuberculosis	
ICD-10: A15–A19	“Tuberculosis”

**Fig. 1:**
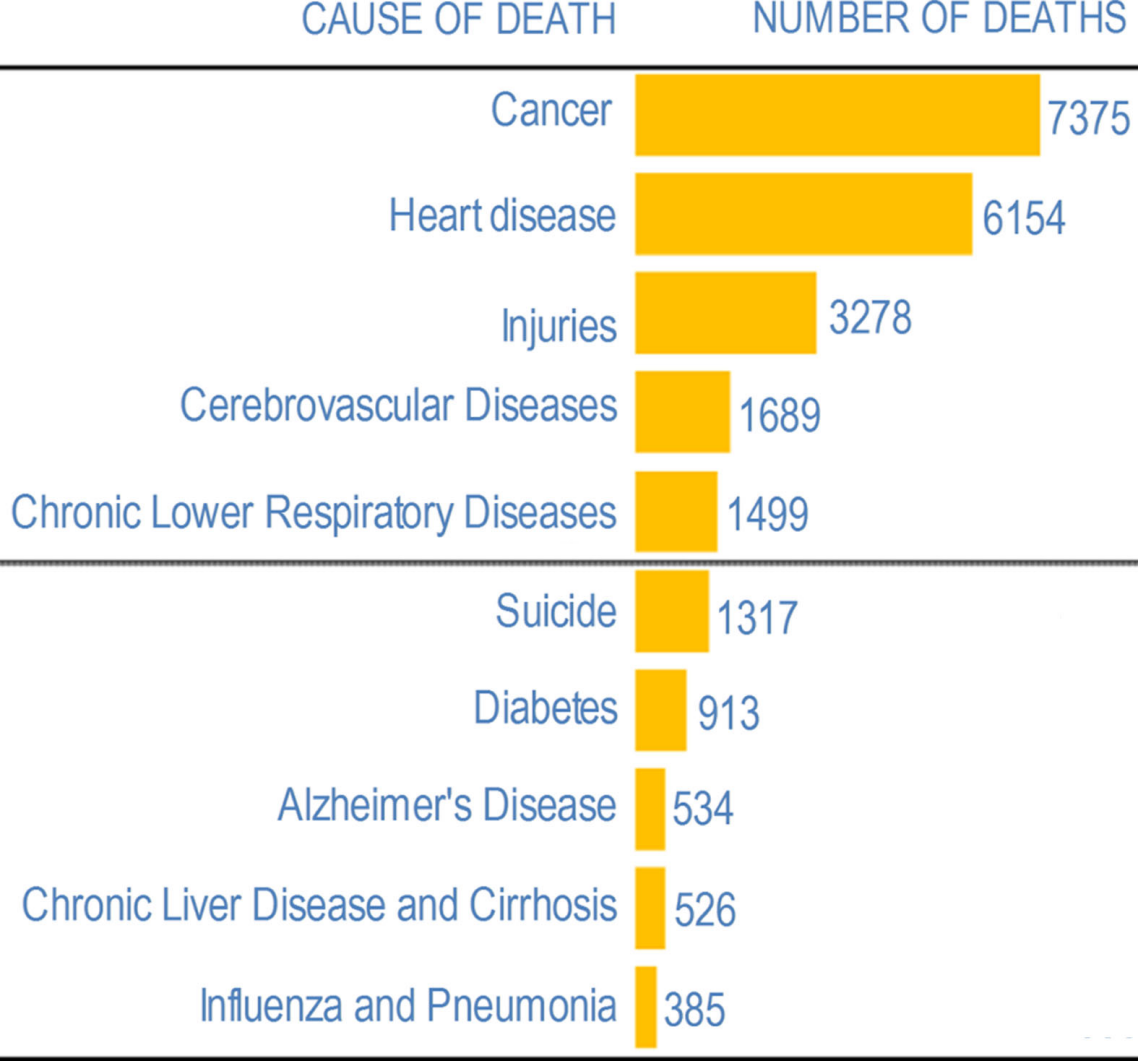
Top 10 causes of mortality in Alaska. Source: Alaska Bureau of Vital Statistics. Last updated: 11/25/2009.

Because this study was intended to inform the research portfolio at the ICHS, the priority health outcomes were the leading causes of mortality and morbidity in Alaska (13). We limited our health outcomes to the 7 leading causes of mortality in the state (Fig. 1), as these 7 categories account for more than 90% of all mortalities in Alaska and Canada. We also included 3 infectious diseases; influenza/pneumonia, sexually transmitted infections (STIs) including HIV, and tuberculosis, as these represent important categories of infectious disease morbidity in Alaska. We consulted the International Classification of Diseases (ICD)-10 (14) and extracted each unique disease term for each relevant set of codes for these 10 priority health outcomes.

The primary search involved entering the geographic search string in the WoK basic search box, and setting search criteria described above, including not limiting the search to the author's geographic affiliation while still including geographic terms relevant to the reported study's location. Then secondary searches were completed by individually entering a disease search string into the “search within results” textbox. This process was repeated 10 times, so that each disease category was individually searched within the complete set of geographical results (Table I). For each search, a single reviewer examined all titles that appeared in the search results. Any title that was not clearly excludable was downloaded to an Endnote database. Each abstract was read completely for exclusion. Abstracts that were not clearly excludable were retained. Complete articles were downloaded in the event of ambiguity over inclusion status. All articles were divided among a review panel composed of the 3 authors and distributed in a way that each article received review by 2 panellists. The panellists reviewed each article to assure inclusion criteria were met, and to assess associations, including study design, between any social determinants and the priority health outcomes. The panel then met and discussed discrepancies. All disputes were settled unanimously through discussion.

## Results

**Fig. 2:**
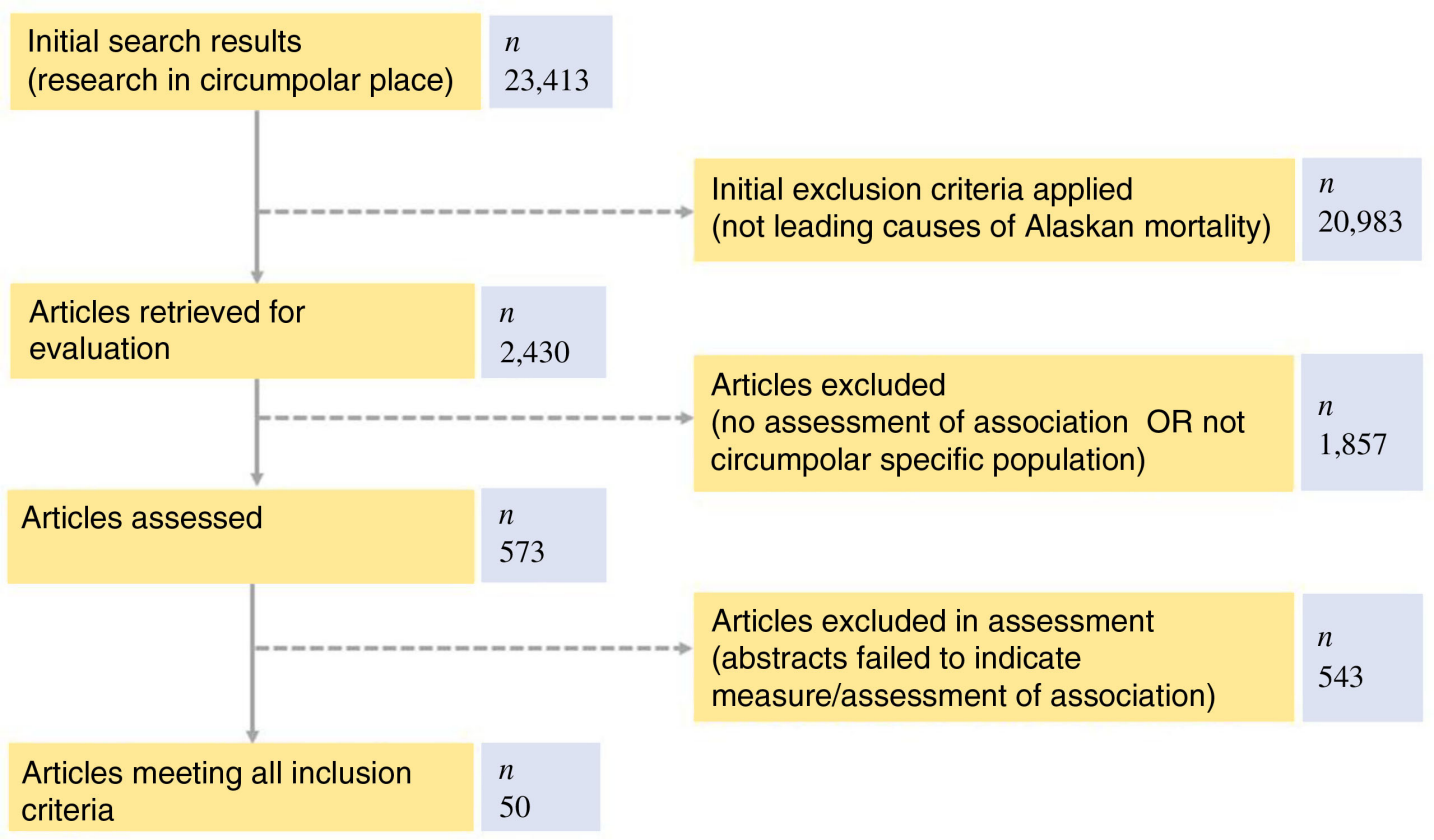
Process for identifying articles for review inclusion.

The initial geographic search had 24,413 hits returned. Secondary searches included only 2,430 titles. Of those, only 573 abstracts remained for downloading. In the end, 30 (Table III) articles fit inclusion criteria. Figure 2 details the article identification process.

The 2011 review identified studies that explicitly assessed associations between 8 determinants and one or more of our 10 priority health outcomes: addiction, social connectedness, environmental exposures, diet/nutrition/exercise, access to quality health care, access to clean water, sexual and reproductive health, and occupational health and safety. Six of these determinants were identified as the topics of health studies in a similar review of the literature in 2009, along with one other: climate change. The 2 additional determinants identified in 2011 from 2009 were sexual and reproductive health and occupational health and safety. One overlapping category, social isolation, was reclassified as social connectedness. While social isolation results in stress and contributes to disease, social connectedness provides a buffering effect leading to resiliency. Both ends of the continuum are included in the category of social connectedness (or lack thereof). Table II details social determinant categories and their definitions.

**Table II:** Identified social determinant categories and corresponding definitions for 2009 and 2011

Determinant	Definition
Addiction***	Confluence of psychological, social, and biological forces that combine to promote and support compulsive substance use
Social isolation* (reclassified as connectedness)	Relationships (or lack thereof) between individuals and others and the benefits (or detriments) of those relationships for individuals and society
Environmental exposures***	Presence of environmental hazards that adversely affect health or the ecological balances essential to human health
Diet, nutrition and exercise*** (Exercise added in 2011)	Procurement, consumption, and metabolizing nutrients necessary to maintain life and health
Access to quality health care***	Effective health care service utilization
Access to clean water***	Processes, quantity, and quality of water obtained for hygiene and consumption
Global climate change*	Adverse environmental factors induced by rapid changes in the Earth's climate
Sexual and reproductive health**	Sexual and reproductive behaviors and cognitions
Occupational health and safety**	Behaviors and exposures related to economic participation or subsistence activities

* 2009

** 2011

*** 2009 and 2011.

Several articles had overlapping determinants or leading cause of mortality or morbidity associations. The results included only one random controlled trial design. The single cohort study was associated with addiction. Six of the social determinant categories had 4 or more associated articles that included both cross-sectional and descriptive designs. The category of access to clean water and occupational health and safety each had 1 associated article. The research design for the articles included a cross-sectional and a descriptive, respectively. See Table III for other details.

**Table III:** Articles included for review, social determinants and associated outcomes, and study design

Author(s)	Determinant(s)	Outcome(s)	Design
Amirkhanian et al. (15)	Sexual and reproductive health/addiction	HIV/STI	Desc
Amparo et al. (16)	Diet, nutrition and exercise/addiction	Heart/diabetes	C-Sect
Balabanova et al. (17)	Addiction/access quality healthcare	TB	Desc
Bendokiene et al. (18)	Sexual and reproductive health/environmental exposure	Heart	C-Sect
Benson et al. (19)	Access clean water	Diabetes	C-Sect
Bevier et al. (20)	Sexual and reproductive health	Cancer	Desc
Bevier et al. (21)	Sexual and reproductive health	Cancer	Desc
Bonefeld-Jorgensen et al. (22)	Environmental exposure	Cancer	C-Sect
Burks et al. (23)	Connectedness/addiction/access quality healthcare	HIV/STI	Desc
Castaldi et al. (24)	Addiction	COPD	C-Sect
CDC (25)	Occupational health and safety/environmental exposure	Injury	Desc
Cepeda et al. (26)	Connectedness	HIV/STI	Desc
Crump et al. (27)	Sexual and reproductive health	COPD	Desc
Epstein et al. (28)	Diet, nutrition and exercise	Cancer	Desc
Grandjean et al. (29)	Diet, nutrition and exercise/environmental exposure	Diabetes	Desc
Hvid et al. (30)	Access quality healthcare	Suicide	RCT
Jolly et al. (31)	Diet, nutrition and exercise	Heart disease	C-Sect
Katz et al. (32)	Connectedness	Suicide	C-Sect
Kim et al. (33)	Sexual and reproductive health	HIV/STI	Desc
Kissin et al. (34)	Addiction	HIV/STI	Desc
Konradi et al. (35)	Addiction/diet, nutrition and exercise/connectedness	Heart/diabetes	Desc
Kral et al. (36)	Addiction/connectedness	Suicide	Desc
Martin et al. (37)	Addiction	HIV/STI	Cohort
Mohatt et al. (38)	Connectedness	Suicide	Desc
Orell et al. (39)	Diet, nutrition and exercise	COPD	C-Sect
Van Hemelrijck et al. (40)	Diet, nutrition and exercise	Cancer	Desc
Walczewska et al. (41)	Connectedness	Heart/diabetes	C-Sect
Wang et al. (42)	Diet, nutrition and exercise/addiction	Heart	Desc
Wood (43)	Addiction	Injury	C-Sect
Zhan et al. (44)	Access quality healthcare/sexual and reproductive health	HIV/STI	Desc

**Fig. 3:**
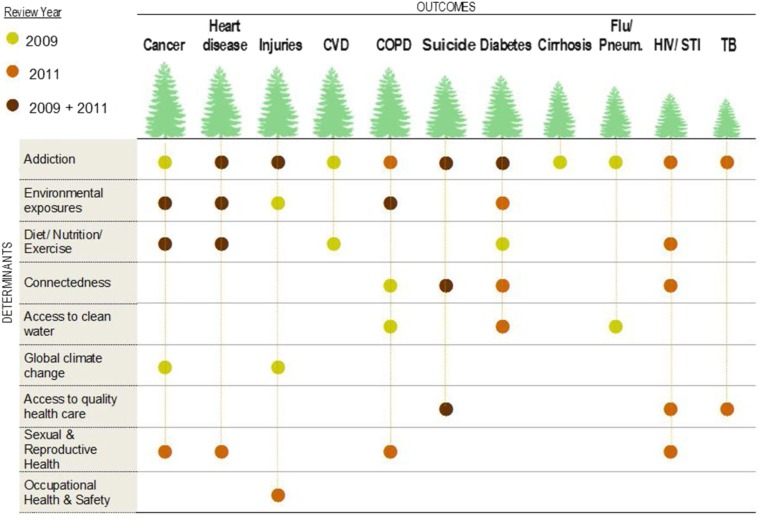
Comparison of findings from 2009 and 2011 Reviews showing 11 disease and 9 social determinant categories.

Comparison with 2009 review results showed 38 points of connection between 11 disease categories and 9 social determinant categories (Fig. 3). The most common social determinant of the health outcomes assessed in this study is addiction. Addiction is associated with all 11 disease outcomes. Of the 38 points of association 29% are represented in both 2009 and 2011, while 32% are represented only in 2009 literature, and 40% are represented only in 2011. Overlaps occur in studies of cancer, heart disease, unintentional injuries, chronic obstructive pulmonary disease, suicide and diabetes. Diabetes had the greatest increase in articles examining new social determinants, while heart disease and suicide remained most consistent with ongoing research, overlapping both review years. Cerebral-vascular disease (stroke) and influenza/pneumonia were the only diseases to see no new research published in 2011. Global climate change was the only determinant established in 2009 with no new research related to the 11 disease outcomes in 2011.

## Discussion

Among the most important questions raised in this series of reviews is the meaning of the observed gaps in publications. On the one hand, the gaps may indicate a lag in the research-publication process. On the other hand, they may indicate areas needing research; areas where additional social determinants may be discovered. It may be that gaps are representative of both issues. For example, social connectedness is a determinant with clear links to heart disease (45).

The above questions expose the limitations of this review. While it is our intention to sample the literature on an ongoing basis, it may be that the bi-annual review process, which we are undertaking primarily due to limitations in personnel time, obfuscates the true range of topics researched. Additionally, the evolving methodology makes precise one-to-one comparisons between annual reviews impossible. This process monitors the pulse of research in the circumpolar north, but by no means provides a comprehensive treatment of the subject.

The greatest use for this review is its utility as a planning tool. By following the current trends in research and publishing, those interested in social determinants of health can better allocate effort and resources. For example, ICHS, in partnership with the Center for Alaska Native Health Research, is planning a project that examines the relation between cultural values and cancer as a social and ecological stressor. Such a project is clearly tied to issues of social connectedness and has the potential to affect cancer incidence and outcomes. These data hold promise for a number of strategic planning processes, none of which is mutually exclusive. Without doubt, as the review process continues to inform our understanding of health and research, our strategies for review and application of findings will continue to change and mature.

Future plans include further incorporation of the 2009 and 2011 results into our strategic plan and research portfolio, as well as replication of the process for presentation at the 2015 International Congress on Circumpolar Health.
